# Maternal Health Equity in Medicaid Accountable Care Organizations: Early Lessons from the Massachusetts Experience

**DOI:** 10.1089/heq.2023.0103

**Published:** 2023-09-13

**Authors:** Laura B. Attanasio, Kimberley H. Geissler

**Affiliations:** Department of Health Promotion and Policy, School of Public Health and Health Sciences, University of Massachusetts Amherst, Amherst, Massachusetts, USA.

**Keywords:** health equity, maternal health, Medicaid, Accountable Care Organizations

## Abstract

There are substantial inequities by race and ethnicity in maternal health care utilization and health outcomes across the perinatal period. As Medicaid covers 42% of births nationally and almost two-thirds of births to Black birthing people, state Medicaid financing and delivery system reforms have substantial scope to impact these inequities. Twenty-one states have implemented Medicaid Accountable Care Organizations (ACOs) at some point since 2015. Using public documents and interviews with ACO administrators, we examine the implications of Massachusetts Medicaid ACOs, implemented in March 2018, for maternal health equity. Although these Medicaid ACOs have the potential to impact maternal health equity, they face many challenges in doing so. We review future steps within Massachusetts Medicaid ACOs and Medicaid programs more generally to incorporate policies that may better address racial and ethnic inequities.

## Introduction

Substantial and persistent racial inequities in maternal health across the perinatal period have been the subject of increasing public health and policy attention. These inequities in maternal health stem from structural racism manifested in interrelated social, environmental, and political factors.^1–3^ Medicaid covers over 40% of births nationally, and disproportionately covers births for birthing people from minoritized racial and ethnic groups. In 2021, Medicaid covered 64% of births to Black individuals and 58% of births to Latinx individuals, versus 28% of births to White individuals.^4^ Current health equity paradigms underscore the importance of organizations in creating and sustaining health inequities.^5^ Comprehensive interventions across the life course are needed to address the complex multilevel barriers to making progress toward health equity.^3,6^ State Medicaid financing and delivery system reforms may represent a structural change in health care delivery with substantial scope to impact racial inequities in maternal health outcomes.

We describe recent reforms to the Massachusetts Medicaid program (MassHealth) under a $1.8 billion Section 1115 waiver demonstration project,^7^ which implemented Accountable Care Organizations (ACOs) for most Massachusetts Medicaid enrollees starting in March 2018, and was renewed in 2023. Although these reforms have the potential to improve maternal health and to address racial disparities in care, a lack of explicit focus on racial inequities and maternal care in activities required by the state Medicaid program was a barrier to improvements.

## ACOs in Medicare and Medicaid

ACOs were designed to incentivize the provision of higher-quality care at lower cost. ACOs are an arrangement in which a group of clinicians provide care for a set of patients and are responsible for the quality and costs of care. The Affordable Care Act created Medicare ACOs, which have been studied extensively for their impacts on cost and quality. Overall, Medicare ACOs have had some positive impacts on process measures of quality and have in some cases achieved modest cost savings.^8^ Limited research on health equity in Medicare ACOs indicates that ACOs with larger proportions of patients from minoritized groups perform worse on quality metrics.^8,9^

An increasing number of states have experimented with ACOs in their Medicaid programs, with 11 states with active Medicaid ACOs in 2023 and 21 states with Medicaid ACOs between 2015 and 2023.^10^ Research on Medicaid ACOs is limited, but some studies to date have found positive impacts on cost and quality outcomes, and a few have found Medicaid ACOs to be associated with reductions in racial or ethnic disparities^11^; none of these studies addressed maternal health disparities. While some studies have addressed impacts of ACOs on birth outcomes and prenatal care utilization.^12,13^ Whether Medicaid ACOs influence maternal health outcomes and equity is unknown, despite the importance of the Medicaid program for the birthing population.

## Implications of Massachusetts Medicaid ACOs for Maternal Health Equity

In this section, we share insights based on document review and interviews conducted in late 2021 and early 2022 with MassHealth ACO administrators as part of a larger project assessing the impacts of Medicaid ACO implementation on maternal health. We interviewed four ACO administrators who had worked with seven unique Medicaid ACOs.

Birthing people were not a population that was explicitly addressed in the goals of the initial waiver creating the ACOs (2017–2023; Table 1). ACO administrators reported simply not focusing on this population in major initiatives during the early implementation period, although some mentioned this as an area of increasing attention over time. Pregnant people with conditions that were a focus of ACOs, such as those with substance use disorder and other behavioral health needs and those experiencing homelessness, may have benefitted from the creation or expansion of services for these populations. Of note, Medicaid ACOs were required to screen enrollees annually for health-related social needs,^7^ which may have identified these needs for pregnant people or better connected enrollees with social services.

**Table 1.** Goals of Initial Massachusetts Medicaid Section 1115 Waiver and Waiver Extension

Goals of the initial wavier (2017–2023)	Goals of the waiver extension (2023–2028)
Enact payment and delivery system reforms that promote integrated, coordinated care and hold providers accountable for the quality and total cost of care	Continue the path of restructuring and reaffirm accountable, value-based care—increasing expectations for how ACOs improve care and trend management, and refining the model
Enact payment and delivery system reforms that promote integrated, coordinated care and hold providers accountable for the quality and total cost of care	Make reforms and investments in primary care, behavioral health, and pediatric care that expand access and move the delivery system away from siloed, fee-for-service health care
Maintain near-universal coverage	Maintain near-universal coverage, including updates to eligibility policies to support coverage and equity
	***Advance health equity, with a focus on initiatives addressing health-related social needs and specific disparities, including maternal health and health care for justice-involved individuals***
Sustainably support safety net providers to ensure continued access to care for Medicaid and low-income uninsured individuals	Sustainably support the Commonwealth's safety net, including ongoing, predictable funding for safety net providers, with a continued linkage to accountable care
Address the opioid addiction crisis by expanding access to a broad spectrum of recovery-focused substance use disorder services	

Sources: Commonwealth of Massachusetts Executive Office of Health and Human Services (EOHHS), Office of Medicaid, “Section 1115 Demonstration Project Amendment and Extension Request,” June 15, 2016.

Commonwealth of Massachusetts Executive Office of Health and Human Services (EOHHS), Office of Medicaid, “Section 1115 Demonstration Project Extension Request,” December 22, 2021.

ACOs, Accountable Care Organizations.

Organizational and contractual challenges to addressing the quality of perinatal care through ACOs may have also blunted any impact of the ACOs on the birthing population. The Medicaid ACO program was oriented around primary care providers (PCPs), with enrollees attributed to a single PCP for contractual purposes of calculating quality and shared savings and losses. PCPs were required to exclusively contract with a single MassHealth ACO. Maternity care clinicians (obstetrician-gynecologist and certified nurse midwives) are not considered PCPs under the ACO structure, and may provide care to patients from multiple ACOs.^14^ This may hinder maternity care clinicians from being fully incorporated into the ACO structure. Administratively, it may also be challenging for ACOs and affiliated PCPs to prospectively identify and track which members are pregnant, particularly for those who are already enrolled and then become pregnant.

In the next waiver extension (2023–2028), addressing health equity, including in maternal health, is one of five specific goals (Table 1). MassHealth will incentivize progress toward this goal by allowing ACOs and some hospitals to earn payments for performance on equity metrics. This will first involve the collection of high-quality data on social risk factors, including race and ethnicity, gender identity, and socioeconomic status, reporting quality metrics stratified by these factors, and finally the reduction of identified inequities.^15^

Through ACOs, enhanced care coordination services will be provided to pregnant individuals with risk factors increasing their chances of an adverse outcome during the perinatal period. While improved data collection is a necessary step toward improving equity, it is not sufficient. A commitment to antiracism in actions by both the Medicaid program and delivery system partners must be present to create meaningful improvement. Currently, the only ACO quality performance measure specific to maternal health is timeliness of prenatal care, which is not a robust measure of health care quality across the perinatal period.^16^

If the slate of quality performance measures relevant to maternal health is not enhanced, this may limit the utility of stratified reporting to enhance maternal health equity.

Some ACOs had already begun to prepare for the collection of social risk factor measures in advance of the waiver extension, such as investing in infrastructure to better capture these data. However, challenges remain. One participant noted that people are more distrustful of the medical system after the COVID-19 pandemic, particularly in communities of color, making the collection of race and ethnicity data more challenging. This participant underscored the importance of training those who are going to be collecting data in best practices for data collection.

## Additional Changes to State Medicaid Programs May Be a Further Opportunity to Improve Maternal Health Equity

In addition to financing and delivery system reforms of Medicaid ACOs, additional changes to state Medicaid programs may provide opportunity to improve perinatal care access and quality in ways that differentially improve care for birthing people from minoritized groups. Improvements include expansions of Medicaid coverage as well as expansions of covered services available to birthing people. As of April 2023, 38 states have extended or are planning to extend postpartum Medicaid coverage from 60 days after birth to 12 months postpartum,^17^ a critical time in which approximately one in nine maternal deaths occur, particularly due to cardiovascular causes that disproportionately contribute to maternal mortality among Black birthing people.^18,19^

Nine states and the District of Columbia include birth doulas as a covered benefit in Medicaid^20^; although far from a panacea, evidence shows that doula coverage may improve health equity.^21^ The Massachusetts Medicaid program is implementing both of these improvements, coverage of doula services and postpartum Medicaid extension, as part of the 1115 waiver extension but not specific to ACOs.

Four states, including Massachusetts, include health-related social needs as a covered Medicaid benefit, including housing and nutrition supports.^22^ Although these benefits only sometimes apply to birthing people during pregnancy and postpartum, intentionally screening for health-related social needs as required in the Massachusetts Medicaid ACOs may be an opportunity to connect birthing people to other government programs such as the Women, Infants, and Children Nutrition Program and Temporary Assistance for Needy Families. Finally, as is clear from the early Massachusetts Medicaid ACO experience, better collection of race and ethnicity data, as well as use of these data to identify and address disparities, may be a way forward for maternal health equity in Medicaid.

## Conclusion

The next 5 years will be a critical period in Massachusetts for understanding whether an explicit stated commitment to advancing health equity, including in maternal health, coupled with policies to encourage collection of race/ethnicity data, examine disparities in outcomes, encourage care coordination, and implementing evidence-based policies such as doulas and postpartum Medicaid extension will be sufficient for turning the tide and improving outcomes during the perinatal period for birthing people from minoritized groups. If the effects of Medicaid ACOs are limited—because they do not provide sufficient incentives for improvement in maternal health or because further multifaceted interventions in the health system and social environment more broadly are required—there may be a loss of opportunity. However, if there is meaningful progress, policy makers may gain a more robust evidence base for Medicaid ACOs and clarity on which factors are most influential in maternal health equity.

## Abbreviations Used

ACOs: Accountable Care Organizations
PCPs: primary care providers

## References

[1] Michener JD. Politics, pandemic, and racial justice through the lens of Medicaid. Am J Public Health [Internet] 2021;111(4):643–646; doi: 10.2105/AJPH.2020.306126 33507819 PMC7958048

[2] Howell E, Ahmed Z. Eight steps for narrowing the maternal health disparity gap: Step-by-step plan to reduce racial and ethnic disparities in care. Contemp Ob Gyn [Internet] 2019;64(1):30–36. 31673195 PMC6822100

[3] Alvidrez J, Castille D, Laude-Sharp M, et al. The National Institute on Minority Health and Health Disparities research framework. Am J Public Health [Internet] 2019;109:S16–S20; doi: 10.2105/AJPH.2018.304883 30699025 PMC6356129

[4] Osterman MJK, Hamilton BE, Martin JA, et al. Births: Final data for 2021. Natl Vital Stat Rep 2023;72(1):1–53. 36723449

[5] Green TL, Zapata JY, Brown HW, et al. Rethinking bias to achieve maternal health equity: Changing organizations, not just individuals. Obstet Gynecol 2021;137(5):935–940. 33831936 10.1097/AOG.0000000000004363 PMC8055190

[6] Cooper LA, Ortega AN, Ammerman AS, et al. Calling for a bold new vision of health disparities intervention research. Am J Public Health [Internet] 2015;105:S374–S376; doi: 10.2105/AJPH.2014.302386 25905830 PMC4455513

[7] The Commonwealth Medicine Research and Evaluation Unit, The Department of Population and Quantitative Health Sciences at The University of Massachusetts Medical School, The Massachusetts Executive Office of Health and Human Services, The Centers for Medicare and Medicaid Services. Independent Evaluation Interim Report Massachusetts Medicaid 1115 Demonstration Extension 2017–2022 [Internet]. 2021. Available from: https://www.mass.gov/doc/1115-demonstration-interim-evaluation-report/download [Last accessed: April 7, 2023].

[8] Spivack SB, Murray GF, Lewis VA. A decade of ACOs in Medicare: Have they delivered on their promise? J Health Polit Policy Law 2023;48(1):63–92. 36112955 10.1215/03616878-10171090

[9] Lewis VA, Fraze T, Fisher ES, et al. ACOs serving high proportions of racial and ethnic minorities lag in quality performance. Health Aff 2017;36(1):57–66. 10.1377/hlthaff.2016.0626 PMC5269600 28069847

[10] States that Reported Accountable Care Organizations In Place | KFF [Internet]. Available from: https://www.kff.org/medicaid/state-indicator/states-that-reported-accountable-care-organizations-in-place/?currentTimeframe=0&sortModel=%7B%22colId%22:%22Location%22,%22sort%22:%22asc%22%7D [Last accessed: April 10, 2023].

[11] Rosenthal MB, Alidina S, Ding H, et al. Realizing the Potential of Accountable Care in Medicaid [Internet]. 2023. Available from: https://www.commonwealthfund.org/publications/issue-briefs/2023/apr/realizing-potential-accountable-care-medicaid?utm_source=alert&utm_mediu… [Last accessed: April 17, 2023].

[12] Harvey SM, Oakley LP, Yoon J, et al. Coordinated care organizations: Neonatal and infant outcomes in Oregon. Med Care Res Rev 2019;76(5):627–642. 29161977 10.1177/1077558717741980

[13] Muoto I, Luck J, Yoon J, et al. Oregon's coordinated care organizations increased timely prenatal care initiation and decreased disparities. Health Aff 2016;35(9):1625–1632. 10.1377/hlthaff.2016.0396 27605642

[14] Cooper MI, Attanasio LB, Geissler KH. Maternity care clinician inclusion in Medicaid Accountable Care Organizations. PLoS One 2023;18(3 March). 10.1371/journal.pone.0282679 PMC9994708 36888632

[15] Executive Office of Health and Human Services, Office of Medicaid. Section 1115 Demonstration Project Extension Request [Internet]. 2021. Available from: https://www.mass.gov/info-details/1115-masshealth-demonstration-waiver-extension-request#final-submission- [Last accessed: April 7, 2023].

[16] Gadson A, Akpovi E, Mehta PK. Exploring the social determinants of racial/ethnic disparities in prenatal care utilization and maternal outcome. Semin Perinatol 2017;41(5):308–317. 28625554 10.1053/j.semperi.2017.04.008

[17] Kaiser Family Foundation. Medicaid Postpartum Coverage Extension Tracker [Internet]. Available from: https://www.kff.org/medicaid/issue-brief/medicaid-postpartum-coverage-extension-tracker/ [Last accessed: April 15, 2023].

[18] Petersen EE, Davis NL, Goodman D, et al. Vital signs: Pregnancy-related deaths, United States, 2011–2015, and strategies for prevention, 13 states, 2013–2017. Morb Mortal Wkly Rep 2019;68(18):423–429. 10.15585/mmwr.mm6818e1 PMC6542194 31071074

[19] MacDorman MF, Thoma M, Declcerq E, et al. Racial and ethnic disparities in maternal mortality in the United States using enhanced vital records, 2016–2017. Am J Public Health 2021:1673–1681. 34383557 10.2105/AJPH.2021.306375 PMC8563010

[20] National Academy for State Health Plans. State Medicaid Approaches to Doula Service Benefits [Internet]. Available from: https://nashp.org/state-medicaid-approaches-to-doula-service-benefits/ [Last accessed: April 18, 2023].

[21] Safon CB, McCloskey L, Ezekwesili C, et al. Doula care saves lives, improves equity, and empowers mothers. State Medicaid programs should pay for it. Health Affairs Forefront [Internet]. Available from: https://www.healthaffairs.org/do/10.1377/forefront.20210525.295915/full/ [Last accessed: April 18, 2023].

[22] McConnell KJ, Rowland R, Nevola A. A Medicaid benefit for health-related social needs. JAMA Health Forum 2023;4(2):e225407. 36800193 10.1001/jamahealthforum.2022.5407

